# Fluid resuscitation in human sepsis: Time to rewrite history?

**DOI:** 10.1186/s13613-016-0231-8

**Published:** 2017-01-03

**Authors:** Liam Byrne, Frank Van Haren

**Affiliations:** 1 Australian National University Medical School, Canberra, Australia; 2 Intensive Care Unit, The Canberra Hospital, Canberra, Australia

## Abstract

Fluid resuscitation continues to be recommended as the first-line resuscitative therapy for all patients with severe sepsis and septic shock. The current acceptance of the therapy is based in part on long history and familiarity with its use in the resuscitation of other forms of shock, as well as on an incomplete and incorrect understanding of the pathophysiology of sepsis. Recently, the safety of intravenous fluids in patients with sepsis has been called into question with both prospective and observational data suggesting improved outcomes with less fluid or no fluid. The current evidence for the continued use of fluid resuscitation for sepsis remains contentious with no prospective evidence demonstrating benefit to fluid resuscitation as a therapy in isolation. This article reviews the historical and physiological rationale for the introduction of fluid resuscitation as treatment for sepsis and highlights a number of significant concerns based on current experimental and clinical evidence. The research agenda should focus on the development of hyperdynamic animal sepsis models which more closely mimic human sepsis and on experimental and clinical studies designed to evaluate minimal or no fluid strategies in the resuscitation phase of sepsis.

## Background

Sepsis is a significant global health problem. The worldwide incidence of sepsis is increasing, with current estimates between 20 and 30 million cases annually [1–5]. The mortality of septic shock, the most severe form of sepsis, continues to be higher than 50% [5, 6]. The cost associated with a single admission of a patient with sepsis has been estimated to be in excess of Euro 20,000 [7, 8]. The treatment of sepsis has not significantly changed over the past 40 years, with the currently used therapies of antibiotics, source control, fluid resuscitation and judicious use of vasopressors recommended in a familiar form in the literature as far back as 1970 [9].

Fluid resuscitation remains the most enduring of sepsis treatments predating even antibiotics. Its beginnings date to the European cholera epidemic of the 1830s, where it was first used to replace the losses associated with cholera diarrhoea [10]. In modern practice, fluid resuscitation has developed to encompass both corrections of absolute and relative hypovolemic states with the additional goal of augmenting cardiac output to restore organ perfusion pressure and improve oxygen transport to cells [5, 11, 12].

Unfortunately, there is no agreed uniform definition of fluid resuscitation in the literature. Fluid administration is not necessarily the same as fluid resuscitation. It is important to differentiate between fluid substitution and volume substitution in intensive care patients, a distinction that has not always been appreciated sufficiently in the design of fluid studies [13]. For the sake of simplicity and consistency, we define fluid resuscitation for sepsis in this review as the administration of intravenous fluids to correct sepsis-induced tissue hypoperfusion. This definition is consistent with the surviving sepsis guidelines and implies a targeted approach to a clinical problem [12].

The following article reviews the rationale for the introduction of fluid resuscitation as treatment for sepsis and highlights a number of significant concerns based on current experimental and clinical evidence.

## Evolution of fluid resuscitation as a therapy for septic shock

R. Hermann was a German chemist working in Moscow during the second cholera pandemic in 1830. He proposed injecting water into the circulation to replace lost fluid after observing haemoconcentration in cholera patients. His clinician colleague Jaehnichen subsequently injected a cholera patient intravenously with 6 oz. of water, which resulted in a notable improvement in the patient’s pulse. However, the patient died 2 h later [14, 15]. When the cholera pandemic arrived in England, in 1831 O’Shaughnessy, on also demonstrating haemoconcentration in cholera patients, recommended intravenous fluids to “restore blood to its natural specific gravity” and “restore its deficient saline matters” [16]. This leads to Latta’s first successful attempts at fluid resuscitation with crystalloid solutions [10, 14–16]. Despite these and other initial reports of success during the pandemic, the practice did not achieve widespread use [10].

Fluid resuscitation was re-discovered some 30 years later as a treatment for severe haemorrhage. In 1864, Goltz was the first to suggest that loss of intravascular volume rather than a loss of oxygen carrying capacity might be the central mechanism of death in haemorrhagic shock [17]. This was supported by the experiments of Konecker and Sander, who found that dogs who were haemorrhaged 50% of their blood volume could be successfully resuscitated with a crystalloid solution [17]. Following these initial experiments, numerous clinical reports described the successful use of intravenous fluids in cases of major surgical and obstetric haemorrhage. Fluid resuscitation then began to be recommended as a treatment for haemorrhage [18–22].

The development of a new paradigm for the condition “shock” accompanied the expansion of the use of intravenous fluids. Prior to the nineteenth century, “shock” remained a concept without a clear definition [23, 24]. It was recognised that many different insults could lead to this common terminal syndrome [23, 24]. Theories on pathogenesis were numerous and varied, and focused mainly around primary nervous system failure or exhaustion of “vital energies” [23, 25]. Parallel developments in medical technology, such as the development of the sphygmomanometer in 1903, led to the ability to reliably and non-invasively measure blood pressure [24]. Experiments by Crile and others showed that injuries such as trauma, manipulation of internal organs and electrocution were able to produce a syndrome of systemic hypotension [25, 26]. On that basis, Mummery and Crile redefined shock as a condition with inadequate systemic blood pressure as its hallmark [24, 27]. Crile’s later experiments demonstrated intravenous saline as one of the few therapies capable of improving blood pressure in shock [27, 28]. By the early 1900s, several articles appeared recommending fluid boluses as an effective therapy for the undifferentiated syndrome of “shock” [29, 30].

The ability to measure cardiac output fundamentally changed the paradigm of shock again. The increased understanding of circulatory pathophysiology allowed the differentiation and description of shock by Blalock into the different subtypes recognisable today [31]. The subtype of vasodilatory shock (vasogenic in the original article) accounts for the dominant phenotype seen in human septic shock and endotoxemia [9, 32–35]. The ability to measure cardiac output led to the observation of an association between the development of a hyperdynamic circulation and survival in both experimental models and clinical septic shock [35–37]. This compelling finding along with the increased lactate and oliguria of sepsis was interpreted as evidence of potentially reversible tissue hypoperfusion [35–37]. The logical response to this was to introduce therapies which increase cardiac output, with the aim to overcome relative hypoperfusion and therefore improve patients’ outcomes [35–37]. This theory of pathogenesis of organ dysfunction and death in sepsis has been the dominant paradigm since its inception in 1960s [38, 39]. However, evidence is accumulating, suggesting this paradigm may be fundamentally incorrect.

## Changing understanding of sepsis and resuscitation

The case for tissue hypoperfusion in hyperdynamic sepsis was based on the occurrence of an increased lactate, oliguria and delivery-dependent oxygen consumption, with each finding attributed to occur due to inadequate blood flow. It was then assumed that fluid resuscitation would produce clinically relevant improvements in cardiac output able to reverse pathological tissue hypoperfusion. This example of linear clinical reasoning based on physiology is likely to be overly simplistic, and each element can be challenged by current evidence.

### Increased lactate in sepsis

Tissue hypoperfusion leading to tissue hypoxia was thought to be the dominant mechanism accounting for the increased lactate seen in sepsis [37, 40, 41]. This theory has been challenged by the following observations. Direct tissue oximetry in hyperdynamic sepsis failed to show tissue hypoxia. Instead, skeletal muscle partial pressure of oxygen in patients with sepsis was found to be elevated [42, 43]. Similarly, experimental animal models attempting to demonstrate impaired cellular oxidative bioenergetics or abnormal lactate/pyruvate ratios have not demonstrated evidence of critically impaired oxidative metabolism during sepsis [44–47]. More recently, an alternative mechanism has been proposed to explain the increased lactate of sepsis. Aerobic production mediated by increased Na/K^ATPase^ activity has been demonstrated to be a significant contributor to the lactate of sepsis in both human and animal models [44, 48]. Microdialysis measurements in septic patients showed that skeletal muscle lactate was significantly higher than blood lactate levels, indicating that there is net contribution of lactate to the circulation by skeletal muscle, despite an increased skeletal muscle partial pressure of oxygen [42, 43, 48]. This theory was supported by the observation that further lactate production could be abolished and the gradient eliminated by locally inhibiting Na/K^ATPase^ [48].

The production and clearance of lactate during sepsis is a complex, nonlinear and still poorly understood process. The current evidence suggests that elevated blood lactate level during sepsis is not a reliable indicator of tissue hypoxia [49].

### Oliguria in sepsis

Oliguria in patients with sepsis is widely regarded as a surrogate for renal tissue hypoperfusion and often used as a trigger for fluid resuscitation. There is, however, little direct clinical evidence of renal hypoperfusion during sepsis. While the available clinical observational data are limited in both size and quality, where accurate estimates of renal blood flow in sepsis have been reported blood flow has been demonstrated to be markedly increased [50]. Experimental models have demonstrated variable findings with both reduced and increased renal plasma flow reported. It is important to point out that many animal models produce hypodynamic sepsis, which may explain some of this variation. In models of hyperdynamic sepsis, which more accurately mimic the clinical syndrome of human septic shock, increased renal blood flow has been demonstrated [50]. For example, in an ovine model of hyperdynamic septic shock, oliguria occurred despite dramatic increases in both cardiac output and renal artery blood flow [51]. These observations strongly suggest that oliguria is not a function of decreased renal perfusion during sepsis.

More importantly, clinical studies both in sepsis and in other conditions such as burns have shown that fluid resuscitation based on oliguria often has minimal to no effect on urine output and fails to reduce renal dysfunction [52–56].

### Delivery-dependent oxygen consumption

The observation that oxygen consumption can be increased by increasing oxygen delivery in patients with sepsis is often referred to as “delivery-dependent oxygen consumption” [37, 41]. Its presence has been used to infer reversible tissue hypoperfusion and tissue hypoxia in hyperdynamic sepsis [41]. However, the observed increase in oxygen consumption with increased oxygen delivery may be entirely accounted for by coupled effects, such as increased myocardial oxygen consumption and forced increases in renal oxygen consumption, rather than reversal of hypoxia [44]. Secondly, delivery-dependent oxygen consumption has also been observed in the setting of chronically ill patients operating at their baseline. This indicates that the presence of delivery-dependent oxygen consumption may not necessarily indicate critical tissue hypoxia [44, 57]. As Dantzker et al. [58] observed (it) “may represent the normal physiological behaviour of the system rather than an abnormal manifestation of oxygen extraction”.

### Effectiveness of resuscitation therapies on achieving haemodynamic targets

Fluid resuscitation exerts its potentially therapeutic effect by increasing the stressed volume of the circulation leading to increased venous return and cardiac output [59]. Recently, attempts have been made to quantify the potency of fluid administration to achieve this in sepsis. Studies in healthy individuals show increases in blood volume of 25–30% immediately after administration with 10–15% persisting 4 h after the infusion [60]. However, sepsis is known to produce changes in vascular permeability and the glycocalyx structure that may decrease the retention of fluids in the vascular compartment [59]. In an animal model of sepsis and fluid bolus resuscitation, only 0.6% of the fluid bolus remained in the circulation after 20 min [61]. Similar results have been demonstrated in humans with sepsis, with rapid redistribution of a fluid bolus out of the vascular compartment [62–64]. Clinically this translates into very small and short-lived effects of fluid bolus therapy on haemodynamic parameters such as blood pressure, heart rate, cardiac output and urine output [52, 65]. An improved understanding of the physiological effects of fluid administration has led to the development of a revised Starling equation. This new model of transvascular fluid exchange is based on recent research and considers the contributions of the endothelial glycocalyx layer, the endothelial basement membrane and the extracellular matrix [66].

The effect of vasopressors, increasing the stressed volume in the venous circulation, has been relatively overlooked until recently. In patients admitted to intensive care with septic shock, temporary reductions in noradrenaline infusion dose produced corresponding reductions in mean systemic filling pressure and cardiac output [67]. This supports previous observations of increased cardiac output and preload with vasopressor use in patients with septic shock [68, 69]. Interestingly, in a recently published PRCT, the addition of levosimendan to augment cardiac output in patients with sepsis failed to show improvements in clinically relevant outcome parameters [70]. In another study comparing the early use of vasopressin and norepinephrine with or without hydrocortisone, similar clinical outcomes were demonstrated across groups demonstrating a range of possible therapeutic approaches to the haemodynamic management of sepsis [71].

These studies highlight that interventions other than fluid resuscitation could be applied to manipulate haemodynamic variables such as cardiac output in sepsis. The comparative effectiveness of these therapies remains unclear.

## Preclinical evidence for the use of fluid resuscitation in sepsis

Several experimental studies in animals have investigated both the effectiveness of fluid resuscitation in improving the septic shock state and its effect on sepsis mortality.

In animal models of septic shock, fluid resuscitation resulted in modest improvements in a number of physiological variables. The most consistent finding from large animal models of sepsis is that of a short-term improvement in cardiac output associated with fluid resuscitation, with the effect dissipating rapidly after the termination of infusion [72–74]. Similarly, a number of studies have demonstrated modest improvements in gastrointestinal perfusion with fluid resuscitation [73]; however, this finding is not consistent in all the animal studies [74]. These results support the observation that, although gastrointestinal mucosal blood flow is impaired in septic shock, treatment strategies specifically aimed at improving gastrointestinal perfusion such as fluid resuscitation have generally failed to correct mucosal perfusion abnormalities and failed to show improve important clinical endpoints [75].

There are several small and large animal models that demonstrate improvements in mortality with fluid resuscitation. For example, in murine models of both caecal ligation and puncture and endotoxemia, fluid resuscitation has consistently been shown to improve mortality when compared to no treatment and to provide additive benefit to both antibiotics and corticosteroids [76–79]. Similarly, fluid resuscitation improved mortality in porcine and canine models of endotoxemia and peritonitis, respectively [80, 81].

The key problem with these animal studies, as briefly mentioned earlier, is the paucity of models that mirror the clinical presentation of sepsis in humans. The response to both sepsis and endotoxaemia in humans is different to commonly used murine, ovine and porcine models [82]. The dominant clinical form of sepsis in humans appears to be hyperdynamic sepsis, characterised by increased cardiac output and decreased systemic vascular resistance (SVR) [9, 32, 33, 35]. When challenged with intravenous endotoxin, humans also develop a hyperdynamic circulation [34]. This contrasts with the response seen in most large and small animal models, where hypodynamic shock after sepsis or endotoxemia predominates [72–74, 76–78, 82, 83]. These disparities occur from both the design characteristics of the models and the differences in human and experimental animal physiology. For example, both pigs and sheep have large numbers of pulmonary intravascular macrophages that are sensitive to circulating endotoxin. Activation leads to the rapid development of pulmonary hypertension and right heart dysfunction in ovine and porcine models of both sepsis and endotoxemia [82]. A similar clinical presentation of hypodynamic shock is seen in most murine models [76–78]. The dominance of hypodynamic models in the animal literature makes direct extrapolation of the effects of fluid resuscitation to human sepsis particularly problematic. The animal literature supports the conclusion that fluid resuscitation is effective for hypodynamic models of sepsis; however, it yields little insight on its effect in hyperdynamic septic shock.

## Clinical evidence for the use of fluid resuscitation in sepsis

Despite its widespread use, the clinical evidence supporting fluid resuscitation in sepsis remains conflicted. Prior to 2001, its use hinged on physiological justification and a long history of use, as there were no randomised controlled trials (RCTs) that tested fluid resuscitation as an intervention for septic shock. The landmark study of early goal-directed therapy (EGDT) by Rivers et al. and the subsequent single-centre and multicentre follow-up RCTs in China were the first prospective studies suggesting benefit of the use of fluids in septic shock [84–86]. These studies demonstrated the benefits of a multi-intervention approach to the initial management of sepsis. Because fluid resuscitation was a central therapy of EGDT, these studies have been interpreted as strong support for the effectiveness of fluid resuscitation. Accordingly, these studies of EGDT are the cited references for the surviving sepsis guidelines recommending fluid resuscitation as the first haemodynamic intervention for patients in septic shock (1C recommendation—strong recommendation with low-quality evidence) [12]. However, while this body of evidence may support a multifaceted therapeutic approach that includes fluid resuscitation, this does not provide evidence for its effectiveness as an independent therapy. Fluid resuscitation was one of many potentially beneficial interventions that were unevenly distributed between groups, in both frequency and timing of use, including antibiotics, vasopressors, corticosteroids and intensity of medical care [84–86]. Recently, three large RCTs of EGDT have been published [87–89]. In these trials, patients assigned to EGDT received significantly more fluids than patients receiving standard care. EGDT consistently failed to show an improvement in mortality for patients with septic shock, but was associated with more ICU admissions and increased utilisation of ICU resources [90]. These findings do not support the systematic use of EGDT, of which more aggressive fluid resuscitation is a component, in the management of patients with septic shock.

The observational data on the effects of fluid resuscitation in sepsis are conflicting, with studies suggesting equivocal, beneficial and negative effects on mortality.

In one study in 2796 patients across 77 intensive care units to determine the effectiveness of surviving sepsis guidelines recommended therapies, regression modelling showed that fluid challenge for hypotension or elevated lactate had no association with outcome (OR 1.01; 0.73–1.39) [91]. However, several retrospective reviews of septic patients, totalling more than 3000 patients, did report positive associations between increased early resuscitation volumes and improved mortality [92–95]. On the other hand, an increasing number of studies link fluid overload in septic patients to worse outcomes [96–99]. A positive fluid balance has been associated with increased mortality in sepsis in a number of studies, but it remains unclear whether it is a causative factor [6, 100]. For example, in a retrospective analysis of the “vasopressin in septic shock trial” (VASST), an inverse relationship between mortality and fluid balance within the first 12 h was demonstrated [98]. Of great concern were the findings of the “fluid challenges in intensive care” (FENICE) study, a large global inception cohort study [101]. This study showed that methods to predict fluid responsiveness are not used routinely by clinicians when prescribing fluid resuscitation, and safety limits for fluid resuscitation are rarely applied. Importantly, there was no statistically significant difference in the proportion of patients who received further fluids after the previous fluid bolus between those with a positive, with an uncertain or with a negatively judged response to fluids. In other words, patients who were proven to be not fluid responsive continued to receive the same amount of subsequent fluid boluses as did fluid responsive patients. This current practice undoubtedly increases the risk of fluid overload in critically ill patients [102].

Conversely, a conservative fluid strategy may improve patient outcomes, as was shown in the “fluids and catheters treatment trial” (FACTT) [103]. In this study, 1000 patients with acute lung injury were randomised into a conservative and a liberal strategy of fluid management using explicit protocols, which were applied for seven days. Patients in the conservative strategy arm showed significantly improved lung function and shorter duration of mechanical ventilation and intensive care without increasing non-pulmonary organ failures.

Considering the dose–effect relationship and side effects of fluids, fluid therapy should be regarded like other drug therapy with specific indications and tailored recommendations for the timing, type and dose of fluid. Based on this, a conceptual model with four phases of intravenous fluid therapy was recently proposed, which include resuscitation, optimisation, stabilisation and evacuation (ROSE) [104]. Specific strategies for fluid minimisation and de-escalation or de-resuscitation have been reported [105–107]. For example, in a recently published study, in which a protocol restricting resuscitation fluid was compared with standard care after initial resuscitation patients with septic shock showed that patient-centred outcomes all pointed towards benefit with fluid restriction [108]. The concept of de-resuscitation is further strengthened by a post hoc analysis of the RENAL study, which showed that a negative mean daily fluid balance was consistently associated with improved clinical outcomes [109]. A further and more detailed discussion of fluid minimisation, de-escalation or de-resuscitation is outside the scope of this review.

To provide a definitive answer to the crucial question what the true effect of fluid administration in the resuscitation phase of human sepsis is, we need high-level evidence from RCTs that compare fluid resuscitation versus no fluid resuscitation. This approach requires clinical equipoise between these two treatment arms, and therefore perhaps a shift in the way clinicians consider fluid resuscitation. Currently, the only randomised controlled trial of fluid resuscitation in sepsis is the “fluid expansion as supportive therapy trial” (FEAST) [110]. The investigators randomised 3141 (of a planned 3600) children with severe sepsis to receive fluid resuscitation with either 40 ml/kg of 0.9% saline or 4% albumin or no volume resuscitation. The trial was stopped early for harm, demonstrating a 40% increase in mortality in both the volume resuscitation arms. Much has been made with regard to the correct interpretation of these findings [111–113]. It has been suggested that the findings are specific to the unique population with a high incidence of malaria (57%), severe anaemia <5 g/dl (32%) and acidosis (base deficit >8 mmol/l, 51%) with saline and albumin causing disease-specific deterioration and worsening of both anaemia and acidosis [111, 112]. However, the published subgroup analysis does not support these conclusions with similar point estimates for harm independent of prior malaria, baseline haemoglobin and base deficit [110]. Surprisingly, the increase in mortality did not appear to be related to complications of fluid overload but rather to delayed cardiovascular collapse [114].

## Conclusion

Fluid resuscitation is recommended and widely used as the first-line resuscitative therapy for all patients presenting with septic shock. This practice seems mainly based on historical beliefs and an incomplete or incorrect understanding of the pathophysiology of sepsis.

Viewed as a whole, the bench-to-bedside evidence supporting fluid resuscitation as treatment for sepsis remains remarkably weak and highly conflicting. In addition, the indiscriminate use of fluid resuscitation, specifically beyond the initial resuscitation phase, has the potential to cause significant harm.

Although absence of evidence does not equal evidence of absence, one could argue there is an urgent need for better evidence. The research agenda should focus on the development of hyperdynamic animal sepsis models which more closely mimic human sepsis and on experimental and clinical studies designed to evaluate minimal or no fluid strategies in the resuscitation phase of sepsis.

The recent history of intensive care medicine has taught us that overly aggressive attempts to “normalise physiology”, focusing on numbers, may be harmful. Perhaps the most important contribution towards improved outcomes of intensive care patients has been the removal of ineffective and potentially harmful treatments. Until proven otherwise, fluid resuscitation for sepsis fits that description.
